# Bacterial properties changing under Triton X-100 presence in the diesel oil biodegradation systems: from surface and cellular changes to mono- and dioxygenases activities

**DOI:** 10.1007/s11356-014-3668-z

**Published:** 2014-10-08

**Authors:** Karina Sałek, Ewa Kaczorek, Urszula Guzik, Agnieszka Zgoła-Grześkowiak

**Affiliations:** 1 Institute of Chemical Technology and Engineering, Poznan University of Technology, Berdychowo 4, 60-965 Poznan, Poland; 2 Faculty of Biology and Environment Protection, Department of Biochemistry, University of Silesia, Jagiellonska 28, 40-032 Katowice, Poland; 3 Institute of Chemistry and Technical Electrochemistry, Poznan University of Technology, Berdychowo 4, 60-965 Poznan, Poland

**Keywords:** Hexadecane monooxygenases, Catechol 2,3-dioxygenase, Cell surface hydrophobicity, Biodegradation, Fatty acids, Triton X-100, Zeta potential

## Abstract

Triton X-100, as one of the most popular surfactants used in bioremediation techniques, has been reported as an effective agent enhancing the biodegradation of hydrocarbons. However efficient, the surfactant’s role in different processes that together enable the satisfying biodegradation should be thoroughly analysed and verified. In this research, we present the interactions of Triton X-100 with the bacterial surfaces (hydrophobicity and zeta potential), its influence on the enzymatic properties (considering mono- and dioxygenases) and profiles of fatty acids, which then all together were compared with the biodegradation rates. The addition of various concentrations of Triton X-100 to diesel oil system revealed different cell surface hydrophobicity (CSH) of the tested strains. The results demonstrated that for *Pseudomonas stutzeri* strain 9, higher diesel oil biodegradation was correlated with hydrophilic properties of the tested strain and lower Triton X-100 biodegradation. Furthermore, an increase of the branched fatty acids was observed for this strain.

**Electronic supplementary material:**

The online version of this article (doi:10.1007/s11356-014-3668-z) contains supplementary material, which is available to authorized users.

## Introduction

An increasing popularity of biodegradation of petroleum hydrocarbons is inevitably a driving force towards more and more successful methods, possibly increasing the effectiveness of this biological utilization method. Thus, a special emphasis has been put on an addition of surface active agents to the biological systems (Randazzo et al. 2001). These substances can not only increase a very limited water solubility of hydrocarbons (Paria 2008) but also might have an influence on the bacterial surface characteristics (Kaczorek et al. 2013a). However, such changes may strongly depend on the chemical structure of surfactants. Ionic surface active agents were proven to affect the cell surface charge directly and hence the attachment to a hydrocarbon while the effect of nonionic surfactants still stays unclear (Brown and Jaffé 2006).

Triton X-100 belongs to the group of the so called octylphenol polyethoxylate (OPEOn) surfactants (which are widely used in the commercial and industrial products, such as detergents, cosmetics or cleaning agents) and has been proven to be biodegradable by some bacterial strains (Chen et al. 2005). Both its biodegradability and easy commercial accessibility give the opportunity to use Triton X-100 as an enhancing factor for biodegradation of petroleum hydrocarbons.

In spite of many reports proving the effectiveness of hydrocarbon degradation in the presence of surfactants, there have been some papers published being in contradiction to this statement. The possible explanations of such opposite results have already been suggested.

First of all, before application of a surfactant to the particular system, its own biodegradability should be thoroughly tested as it is possible that some microorganisms will use the surface active agent as an initial carbon source, therefore limiting the intended hydrocarbon utilization (Yu et al. 2007). Secondly, the surfactants may interact, either inhibit or activate the oxidizing enzymes (mono- and dioxygenases) responsible for the initial step in the metabolism of hydrocarbons (Aronstein and Alexander 1993). Further issues are related to concentration of the surfactants, their adsorption properties and/or micelle formation as well as their influence on uptake of hydrocarbons by microorganisms (Allen et al. 1999; Wyrwas et al. 2011; Yu et al. 2007).

Bearing in mind these particular problems and factors possibly influencing the biodegradation process carried out in the presence of surfactants, a detailed and precise analysis would be highly required when designing an appropriate biodegradation process.

This study covers the role of the nonionic chemical surfactant, Triton X-100, in the biodegradation of diesel oil. This broad research focuses on the influence of the abovementioned surfactant on different aspects of its work, which together contribute to an effective biodegradation process. The examination includes the cell surface properties (of the tested strains), enzymatic activity of the two main enzymes engaged in degradation pathways of hydrocarbons, zeta potential and fatty acid composition to name a few. All those tests were performed in both the presence and absence of Triton X-100 in biodegradation systems.

## Materials and methods

### Chemicals

Hydrocarbons and other fine chemicals employed in this study were of highest purity grade, produced by Merck (Darmstadt, Germany). Triton X-100 (Sigma-Aldrich, Munich, Germany) is a mixture of octylphenol ethoxylates (OPEOs) with an average degree of ethoxylation equal to 9.5. Diesel oil was purchased from PKN Orlen petrol station and sterilized by filtration before use.

### Microorganism characterization

The strains *Achromobacter* sp. 4(2010), *Pseudomonas stutzeri* strain 9 and *Rahnella* sp. strain EK12 were isolated from hydrocarbon-contaminated soil samples from the Polish Carpathian Mountains. The isolated strains were phenotypically characterized using the standard techniques (gram staining, colony shape, size and colour on nutrient agar plate, catalase and oxidase test, etc.), according to Bergey’s Manual of Determinative Bacteriology (Holt et al. 1994). Bacterial DNA was isolated from the pure culture using the DNA Mini Prep Kit (Qiagen, Venlo, The Netherlands). For 16S rRNA gene amplification, the bacteria-specific primers 8F 5′AGTTTGATCATCGCTCAG 3′ and 1492R 5′GGTTACCTTGTTACGACTT3′ were used (Lonergan et al. 1996). The 16S rRNA gene sequence determined in this study was deposited in the GeneBank database of NCBI under the accession numbers HM246520.1 (*Achromobacter* sp. 4(2010)), JN006140.1 (*P. stutzeri* strain 9) and JQ409469 (*Rahnella* sp. strain EK12).

### Biodegradation test

Diesel oil biodegradation was performed in 250-mL Duran-Schott bottles containing 50 mL of mineral medium. The medium used in experiments consisted of (g L^−1^) Na_2_HPO_4_·2H_2_O 7.0, KH_2_PO_4_ 2.8, NaCl 0.5, NH_4_Cl 1.0, MgSO_4_·7H_2_O 0.01, FeSO_4_·7H_2_O 0.001, MnSO_4_·4H_2_O 0.0005, ZnCl_2_ 0.00064, CaCl_2_·6H_2_O 0.0001, BaCl_2_ 0.00006, CoSO_4_·7H_2_O 0.000036, CuSO_4_·5H_2_O 0.000036, H_3_BO_3_ 0.00065, EDTA 0.001 and HCl 37 % 0.0146 mL L^−1^. The pH of the medium was 7.2. The concentrations of diesel oil in all biodegradation experiments were 1 % (*w/v*). The influence of Triton X-100 on diesel oil biodegradation was analysed using its 120 mg L^−1^ concentrations. The experiment samples contained diesel oil, a culture medium, a few milliliters of bacterial stock cultures (to reach an OD of ca. 0.1) and the surfactant apart from reference samples (samples with no Triton X-100). Each experiment was repeated three times, and values of biodegradation were calculated as a mean value out of three flasks to attain the accuracy of ±5.4 %. The total mass of hydrocarbon residues was determined using the “standard method for gravimetric determination of hydrocarbons” (PN-86 C-04573/01; Kaczorek 2012). The final results were calculated with respect to blank samples (hydrocarbon with medium without microorganisms).

### Liquid chromatography–tandem mass spectrometry analysis of octylphenol ethoxylates

The chromatographic system UltiMate 3000 RSLC from Dionex (Sunnyvale, CA, USA) was used in this step of assays. Five microliters of samples were injected into a phenyl-hexyl column (50 × 3 mm I.D.; 1.8 μm) from Agilent Technologies (Santa Clara, CA, USA). The mobile phase employed in the analysis consisted of 5 · 10^−3^ mol L^−1^ ammonium acetate in water and methanol at a flow rate of 0.3 mL min^−1^ at 35 °C. Triton X-100 was analysed using gradient elution starting from 70 % of methanol changed to 95 % of methanol in 3 min and maintained at 95 % for 5 min. A pre-run time of 4 min was done before the next injection. The chromatographic system was connected to the API 4000 QTRAP triple quadrupole mass spectrometer from AB Sciex (Foster City, CA, USA). The LC column effluent was directed to the electrospray ionization source (Turbo Ion Spray). The Turbo Ion Spray source operated in a positive ion mode. The octylphenol ethoxylates were analysed in multiple reaction monitoring mode. The dwell time for each mass transition was set to 50 ms. The following settings for the ion source were used: curtain gas 20 psi, nebulizer gas 40 psi, auxiliary gas 40 psi, temperature 350 °C, ion spray voltage 4500 V and declustering potential 50 V. The collision gas was set to medium. The detected mass transitions and specific parameters for each analyte are summarized in Table 1.

**Table 1:** Parameters of mass spectrometric detection characteristic to particular analytes (*MRM* multiple reaction monitoring). OPEO1-19, octylphenol ethoxylates, containing 1 to 19 ethoxy groups

Triton X-100	MRM transitions (precursor ion m/z→product ion *m*/*z*)	Collision energy [V]
OPEO1	268.2→113.0	13
OPEO2	312.2→183.0	9
OPEO3	356.3→227.0	17
OPEO4	400.3→272.0	21
OPEO5	444.3→316.0	25
OPEO6	488.4→360.0	26
OPEO7	532.4→133.0	33
OPEO8	576.4→133.0	35
OPEO9	620.4→133.0	37
OPEO10	664.5→133.0	38
OPEO11	708.5→133.0	40
OPEO12	752.5→133.0	41
OPEO13	796.5→133.0	42
OPEO14	840.6→133.0	46
OPEO15	884.6→133.0	46
OPEO16	928.6→133.0	49
OPEO17	972.6→133.0	52
OPEO18	1016.7→133.0	53
OPEO19	1060.7→133.0	55

### Surface properties of tested bacterial strains

All tested strains were grown in mineral medium on different carbon sources: diesel oil (1 % *w/v*), Triton X-100 at different concentrations (6, 60, 120, 240 and 360 mg L^−1^) and their mixtures with diesel oil at 25 °C with stirring. Cells used in analyses were in the exponential growth phase.

#### Cell surface hydrophobicity (CSH)

The cell surface hydrophobicity of bacterial strains was determined using a modified method of microbial adhesion to the hydrocarbon (Górna et al. 2011). The optical density of biomass suspension was measured at 600 nm on the UV-Visible Spectrophotometer Shimadzu (Shim-Pol, Izabelin, Poland), using heptane as the model hydrocarbon. Each experiment was repeated three times, and results for cell surface hydrophobicity were calculated as a mean value of three samples to attain the accuracy of ±1.8 %. Microbial adhesion to hydrocarbon was calculated as

Hydrophobicity (%) = (1–OD_600_ of aqueous phase after mixing with hexadecane/OD_600_ of initial aqueous phase) 100.

#### Zeta potential

The zeta potential was determined by measurements of cell electrophoretic mobility using the ZetaPlus instrument (Brookhaven Instruments Co., Holtsville, NY, USA) and was calculated from the Smoluchowski equation (Miyake et al. 1990).

#### Adsorption parameters of Triton X-100

The equilibrium surface tension was performed using the du Noüy ring technique with the Krüss K12 tensiometer with a platinum ring. The experiments were performed at 21 ± 1 °C.

The surface tension data can be fitted by adsorption equations.

From a physicochemical point of view, it is suitable to use the Szyszkowski equation (Chattoraj and Birdi 1984):

1 $$ {\gamma}^{Sz}={\gamma}_0\left[1-B \ln \left(\frac{C}{A}+1\right)\right] $$

where *γ*_0_ is the surface tension for the distilled water, *A* and *B* are the adsorption coefficients. By using Eq. (1) and introducing the term ∂*γ*/∂*c* into the Gibbs isotherm:

2 $$ \varGamma =-\frac{1}{RT}\cdot \frac{d\sigma }{d \ln C} $$

where *Γ* is the surface excess, *R* gas constant and *T* temperature; Eq. (3) is obtained for the surface excess in the case of the nonionic system:

3 $$ {\varGamma}^{Sz}=\frac{\gamma_0BC}{RT\left(C+A\right)} $$

The adsorption coefficients of the Szyszkowski isotherms *A* and *B* can be used to estimate the Gibbs free energy of adsorption (*ΔG*_*ads*_) and the surface excess at the saturated interface (*Γ* ^∞^).

4 $$ \varDelta {G}_{ads}=-RT \ln A $$

5 $$ {\varGamma}^{\infty }=\frac{\gamma_0B}{RT} $$

#### Fatty acid extraction and analysis

The cellular fatty acids were extracted from cells grown on (1) a nutrient broth, (2) mineral salt medium supplemented with diesel oil, (3) mineral salt medium supplemented with Triton X-100 and (4) mineral salt medium supplemented with Triton X-100 and diesel oil. Diesel oil was added to 1 % concentration and surfactant at 120 mg L^−1^. Bacterial cells were harvested by a centrifugation (8000 *g*) at 4 °C for 20 min and then washed twice with 0.85 % NaCl to remove residues of the culture medium. Further fatty acid isolation and identification were conducted following the MIDI-MIS method according to Sasser (1990). The fatty acid methyl esters (FAMEs) analysis was performed using an HP 5890 gas chromatograph (Hewlett Packard, Rolling Meadows, IL, US) equipped with an HP 25 m × 0.2 mm cross-linked methyl-silicone capillary column. The initial oven temperature was 170 °C, increased every 5 °C min^−1^ to 260 °C and then every 40 °C min^−1^ and eventually held constant at 320 °C for 1.5 min. Helium was used as the carrier gas. FAMEs were identified using Sherlock software (TSBA library, version 3.9, Microbial ID, Newark, NJ, USA) based on the actual calibration retention times run prior to sample analysis.

### The activity of hexadecane monooxygenase and catechol 2,3-dioxygenase in cell free extracts

#### Preparation of cell free extracts

The cell free extracts were prepared as described previously (Sałek et al. 2013). The procedure was performed for the cells of exponential growth phase and covered double centrifugation, washing with a potassium phosphate buffer at pH 7.2 and resuspension of cells in the same buffer. Then, the cell disruption was done by an ultrasonic disintegration followed by a centrifugation (50 min, 13,000×*g*, 4 °C) in order to remove the unbroken cells and the debris. The obtained supernatant was used for further assays.

#### Determination of hexadecane monooxygenase and catechol 2,3-dioxygenase

The activity of hexadecane monooxygenase was determined according to Iwaki et al. (2006). Briefly, the enzyme activity was measured spectrophotometrically by monitoring the decrease in the absorbance of NADH at 340 nm. The standard mixture of total volume 1 mL contained potassium phosphate buffer (pH 7.2), 4 mM NADH, 44 μM FAD, deionized water and the cell free extract. The reaction was started by the addition of 3 μL of hexadecane to the reaction mixture. Specific activities were expressed as unit per milligram of protein.

The activity of catechol 2,3-dioxygenase was determined spectrophotometrically by monitoring the formation of 2-hydroxymuconic semialdehyde at 375 nm (*ε*_375_ = 36,000 M^−1^ cm^−1^) as described by Wojcieszyńska et al. (2011). The standard mixture of total volume 1 mL contained 50 mM catechol, potassium phosphate buffer (pH 7.2) and the cell free extract.

The protein concentrations were determined by the method of Bradford (1976) using lisozyme as a standard.

## Results and discussion

### Diesel oil and Triton X-100 biodegradation

**Fig. 1:**
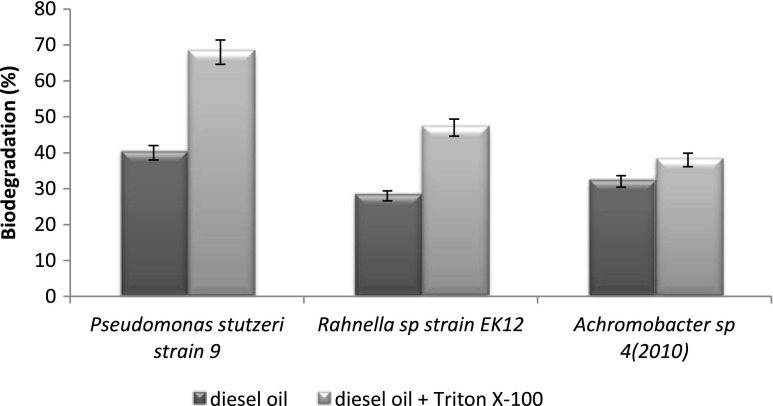
Diesel oil biodegradation by three bacterial strains, *Pseudomonas stutzeri* strain 9, *Rahnella* sp. strain EK12 and *Achromobacter* sp. 4(2010), and the influence of 120 mg L^−1^ Triton X-100 on biodegradation. The process was carried out at 25 °C for 7 days. Results have absolute (100 %) quantitative value

In this study, a diesel oil biodegradation by three bacterial strains, *Achromobacter* sp*.* 4(2010), *P. stutzeri* strain 9 and *Rahnella* sp. strain EK12, after 7 days of experiments was estimated and was followed by the assays of biodegradation in the presence of Triton X-100 (Fig. 1). The laboratory tests with different concentrations of surfactants showed that diesel oil biodegradation was the most effective when 120 mg L^−1^ of Triton X-100 was used. Diesel oil biodegradation depends not only on the kind of surfactant but also its quality. Therefore, before putting them into the system, the amount of surface active agents should be determined. The use of surfactant in biodegradation processes have to be not only cost effective but also ecologically safe.

**Fig. 2:**
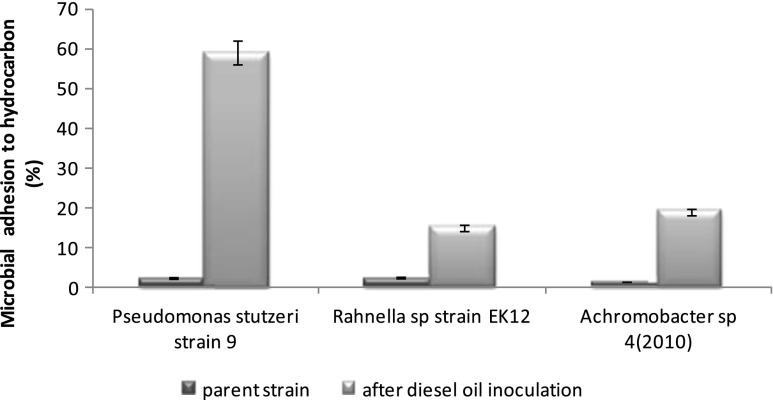
Microbial adhesion to hydrocarbon of three tested bacterial strains, *Pseudomonas stutzeri* strain 9, *Rahnella* sp. strain EK12 and *Achromobacter* sp. 4(2010) after diesel oil inoculation compared to parent strain. The process was carried out at 25 °C for 7 days. Results have absolute (100 %) quantitative value

The biodegradation of diesel oil was the highest for *P. stutzeri* strain 9 (40 %). In the two other systems, the biodegradation reached 28 % for *Rahnella* sp. strain EK12 and 32 % for *Achromobacter* sp*.* 4(2010). The comparison of biodegradation with the cell surface properties in the diesel oil systems showed that higher biodegradation was accompanied by higher cell surface hydrophobicity (Fig. 2). Addition of Triton X-100 to the diesel oil system caused an increase of biodegradation rates in all tested systems. A significant difference was observed for the *P. stutzeri* strain 9, where the biodegradation of diesel oil enhanced with Triton X-100 was higher by 70 %. In this case, an introduction of the surfactant proved to be an effective resolution to enhance the biodegradation. Considering the other two strains and results obtained for their systems, only *Rahnella* sp*.* strain EK12 system showed noteworthy results, while for *Achromabacter* sp. 4(2010), the effect of surfactant was barely noticeable.

**Fig. 3:**
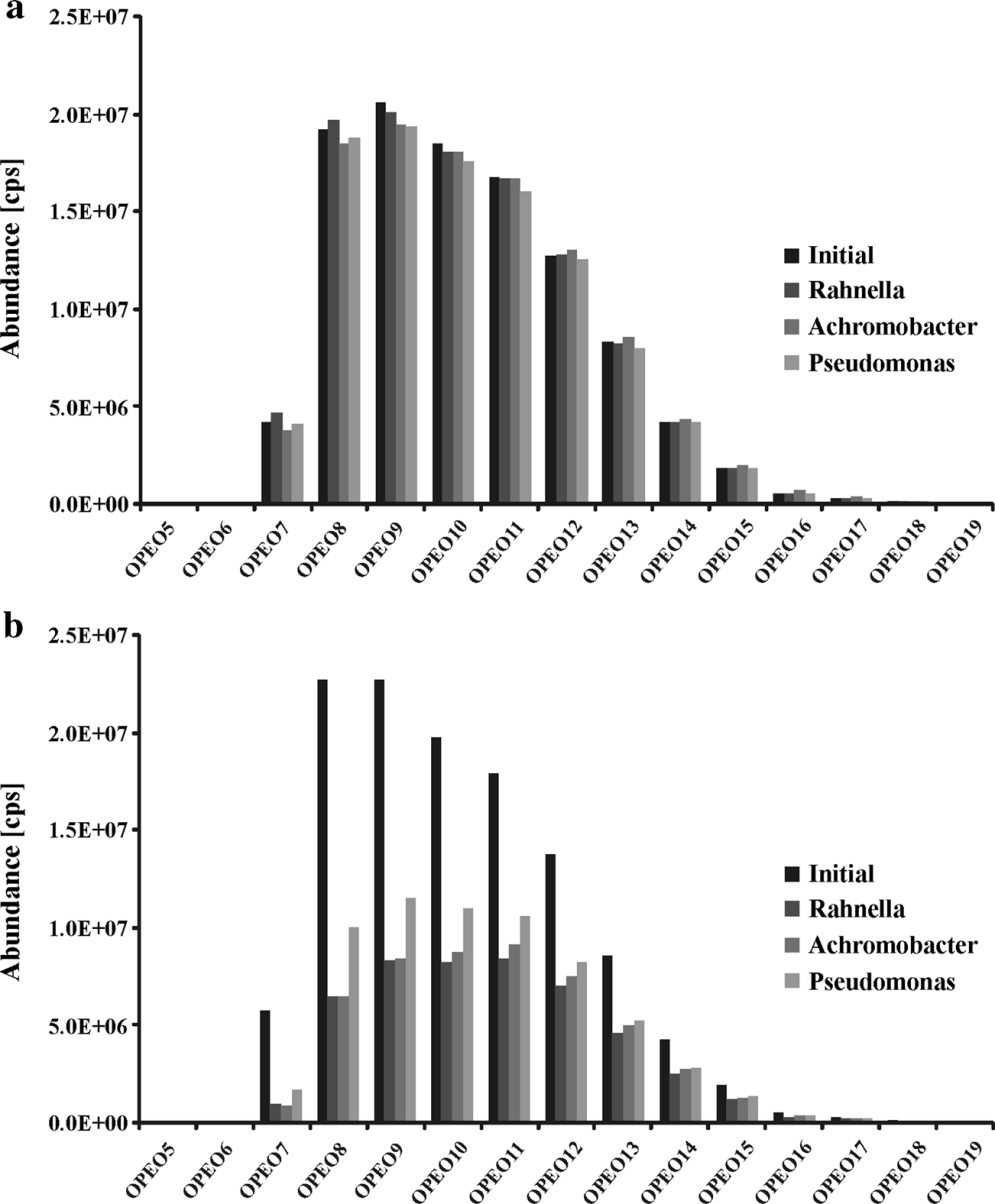
MS ion abundances obtained for Triton X-100 subjected to the biodegradation tests. OPEO5-19, octylphenol ethoxylates, containing 5 to 19 ethoxy groups. Two types of test are without diesel oil (**a**) and with diesel oil added to the sample (**b**). Initial mean results obtained for the start of the tests. *Rahnella* sp. strain EK12, *Achromobacter* sp. 4(2010) and *Pseudomonas stutzeri* strain 9 mean results obtained after 7 days of the biodegradation with or without diesel oil for *Rahnella* sp. strain EK12, *Achromobacter* sp. 4(2010) and *Pseudomonas stutzeri* strain 9, respectively

Our study indicates that Triton X-100 could enhance a diesel oil biodegradation, although many authors also observed its inhibitory effect on the process (Yang et al. 2007). Triton X-100 could also have a positive effect on the removal of many hazardous compounds. According to Mohanty and Mukherji (2012), the use of this surfactant caused an increased biodegradation of non-aqueous phase liquid (NAPL) by the two tested strains. What is more, the authors observed cell surface hydrophobicity (CSH) increasing and zeta potential decreasing during this process. Remarkably, other observations became apparent throughout the experiments conducted in our laboratory; it was found that the biodegradation of Triton X-100 was influenced by the addition of diesel oil (Fig. 3). Practically, no biodegradation of Triton X-100 was found without diesel oil in the samples. The same amount of Triton X-100 was found after 7 days of biodegradation with *Rahnella* sp*.* strain EK12 as in the initial day of the test. Only 1.4 and 3.4 % of Triton X-100 was biodegraded by *Achromobacter* sp*.* 4(2010) and *P. stutzeri* strain 9, respectively. On the contrary, the presence of diesel oil led to some significant biodegradation of surfactants for all tested bacterial strains. Biodegradation of Triton X-100 after 7 days in the presence of diesel oil reached 59, 57 and 47 % for *Rahnella* sp*.* strain EK12, *Achromobacter* sp*.* 4(2010) and *P. stutzeri* strain 9, respectively. This effect was observed mainly for the octylphenol ethoxylates with short ethoxy chains. It could be easily noticed, as no chain shortening was found in the tests. It is also worth emphasizing that *P. stutzeri* strain 9 is the least effective among the tested strains when considering the biodegradation of Triton X-100 with diesel oil, although at the same time, it was capable to cause the highest biodegradation of diesel oil in the same test. A higher result of biodegradation of Triton X-100 by the two other strains was connected with a lower biodegradation of diesel oil. To summarize, a coexistence of diesel oil and a surface active agent improved the biodegradation of both these substances. Also, consumption of diesel oil lowered the biodegradation of surface active agent and vice versa.

#### Cell surface hydrophobicity and zeta potential

Owing to the presence of ionized forms of phosphoryl and carboxylate groups localized on the outer membrane of cells hence building and extracellular environment, bacteria usually possess a negative surface charge (Wilson et al. 2001). And as the outer membrane properties play an essential role in bacterial adhesion and exchange processes, the zeta potential (defined as the electrophoretic mobility of cells in an electric field) may be helpful in assessing and/or calculating the overall cell surface polarity/net cell surface charge (Wilson et al. 2001). Combined with the hydrophobicity, these results can possibly indicate the overall mechanisms of cell attachment and behaviour in different media.

**Fig. 4:**
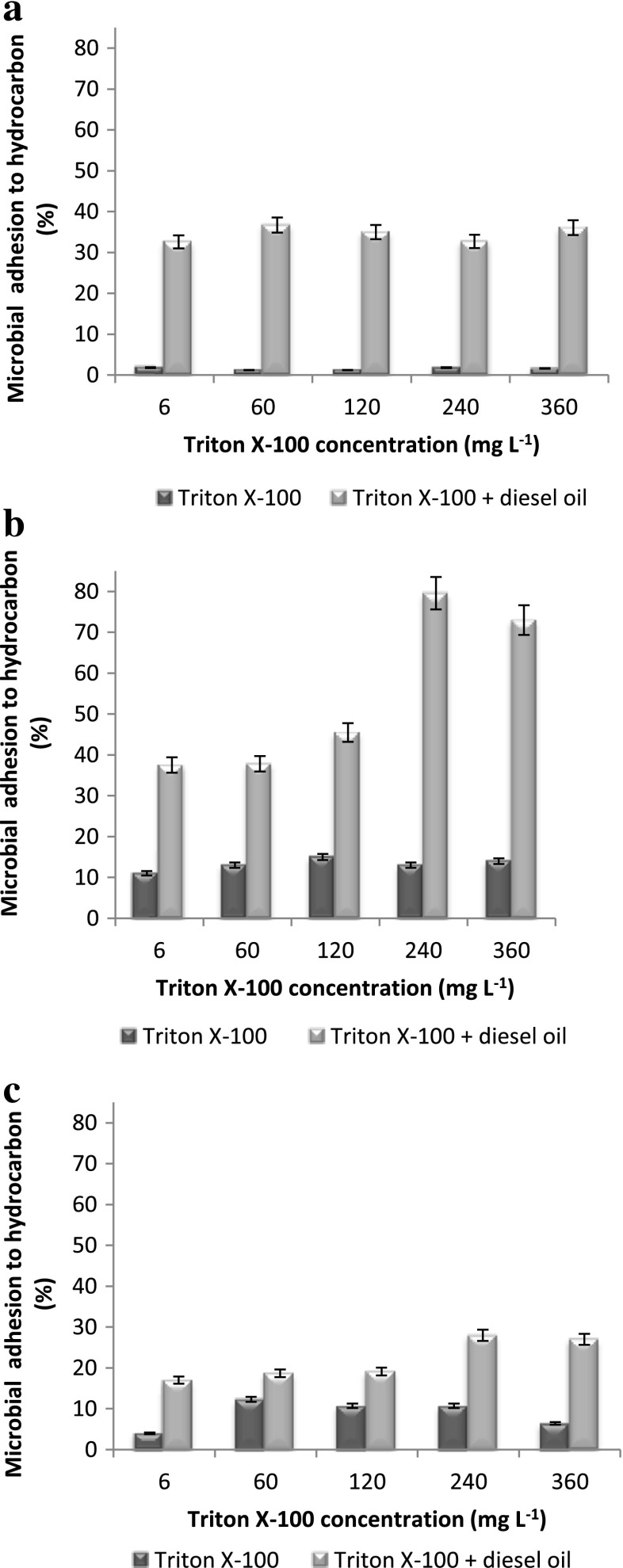
Microbial adhesion to hydrocarbon of *Pseudomonas stutzeri* strain 9 (**a**), *Rahnella* sp. strain EK12 (**b**) and *Achromobacter* sp. 4(2010) (**c**) cultivated in Triton X-100–diesel oil and diesel oil systems. The concentration of diesel oil was 2 % (*w/v*) and surfactant 6, 60, 120, 240 and 360 mg L^−1^. The process was carried out at 25 °C for 7 days. Results have absolute (100 %) quantitative value

The influence of diesel oil inoculation on cell surface hydrophobicity (CSH) was noticed. The highest CSH was observed for *P. stutzeri* strain 9 (Fig. 2). However, addition of Triton X-100 in different concentrations to the diesel oil system caused a decrease in the cell surface hydrophobicity of this strain. A different situation was observed for the following strains: *Rahnella* sp. strain EK12 and *Achromabacter* sp. 4(2010). The tested systems were dominated by hydrophobic cells (Fig. 4).

**Fig. 5:**
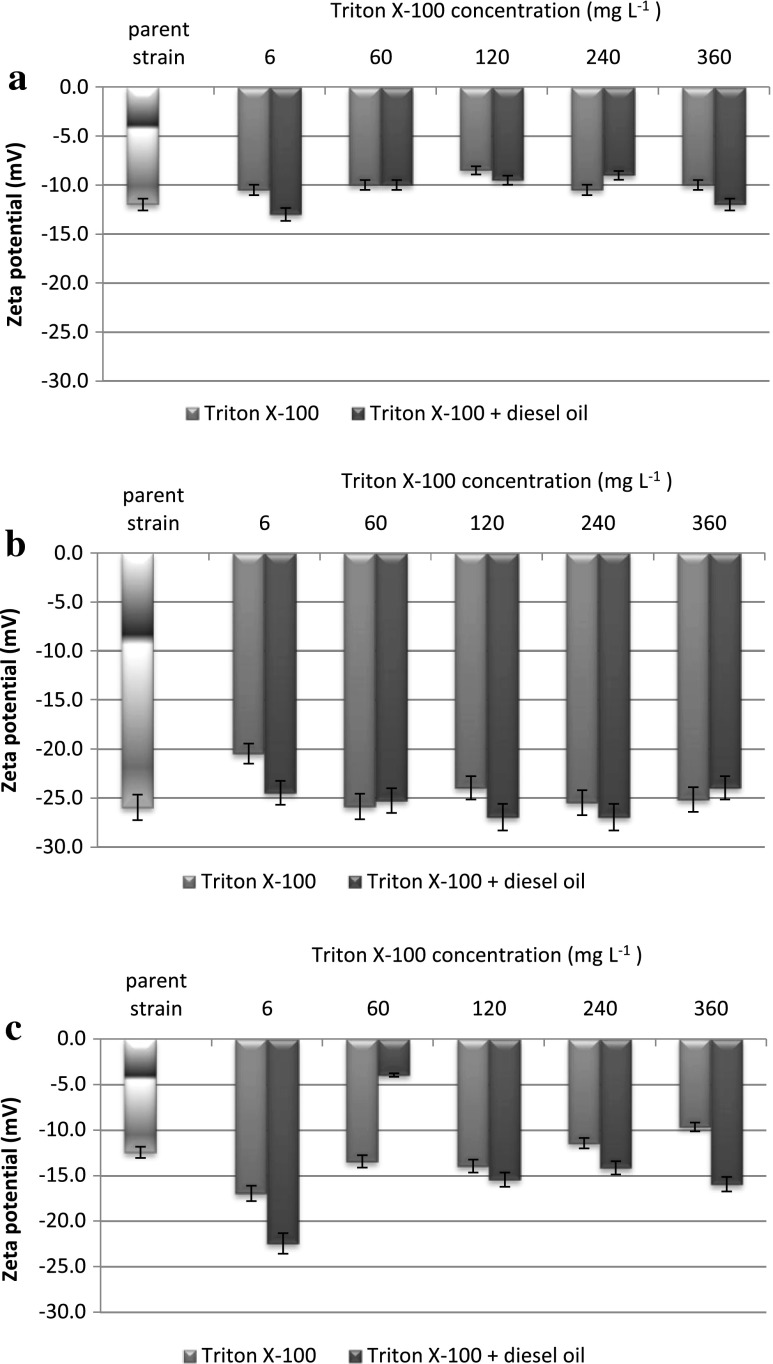
Zeta potential of *Achromobacter* sp. 4(2010) cells (**a**), *Pseudomonas stutzeri* strain 9 cells (**b**) and *Rahnella* sp. strain EK12 cells (**c**) in diesel oil and diesel oil–Triton X-100 systems. Surfactant concentrations are 6, 60, 120, 240 and 360 mg L^−1^. Results have absolute (100 %) quantitative value

The present research in this field covered the zeta potential measurements of three bacterial strains in three basic forms: (1) a pure form, named “parent strain” and meaning the initial zeta potential of the bacterial cells in a standard salts medium; (2) strain + Triton X-100 form, indicating the changes of bacteria properties under different concentrations of surfactant with no additional hydrocarbon; and (3) strain + Triton X-100 + diesel oil form—the samples taken from the biodegradation tests and showing the reaction of cells surface properties in those specific systems. The results presented in Fig. 5 underline the most visible difference—the initial zeta potential values (“parent strain” forms) for all the strains. The highest zeta potential (−12 mV) was detected for *Achromobacter* sp. 4(2010), while the lowest result characterized the *P. stutzeri* strain 9. When exposed to Triton X-100, *Achromobacter* sp. 4(2010) did not present any valuable changes in zeta potential; the values were almost negligible in comparison to the parent strain and were within the standard error. The same observation can be implemented to the system with a diesel oil (a third form as indicated previously in this chapter). Similar behaviour of another strain, *P. stutzeri* strain 9, was likewise observed. However, that trend did not cover the last tested strain *Rahnella* sp. strain EK12 where the most noticeable changes could be distinguished. Firstly, the 6 mg L^−1^ of a surfactant in the system led to the most significant change of the zeta values (from −12.5 mV to almost −17.5 mV); and when the same system with diesel oil was considered, the decrease was even more explicit reaching −23 mV. The other concentrations of Triton X-100 did not alter the cells’ potential, only in the presence of diesel oil in the systems slightly lowered the values. The visible and rather unexpected change was observed for the 60 mg L^−1^ of Triton and with the presence of diesel oil. A further, general consideration of the zeta potential results brings some general conclusions: first of all, there are no meaningful results that would show Triton X-100 direct interactions with the cells. Secondly, even the presence of diesel oil would not diversify the overall trends observed for the strain + Triton X-100 forms. To conclude, the zeta potential parameters did not significantly change when the surfactant and/or the surfactant and diesel oil systems were analysed, that leads to the general comment that in this particular case the effectiveness of biodegradation did not depend on the electrophoretic mobility of particles (bacterial cells) and no correlation could be observed.

### Adsorption parameters of Triton X-100

The Szyszkowski equation was used to calculate the adsorption parameters of Triton X-100 in two biological systems with *P. stutzeri* strain 9 and *A. denitrificans* sp. 4(2010). The surface excess at the saturated interface, minimum surface occupied by a statistical molecule and the free energy of adsorption were determined (Table 2). Triton X-100 had a larger surface activity in biological systems than in a medium solution. In those systems, there was the biggest tendency to adsorption observed; meanwhile, the free energy of an adsorption was the lowest. Among the tested biological systems, a larger surface activity was observed for *P. stutzeri* strain 9 (−∆*G*_*ads*_ = 17.5).

**Table 2:** Adsorption parameters of Triton X-100 in a mineral salt solution and in a biological system

Parameter	Unit	Triton X-100 (medium solution)	Triton X-100 (biological system, *Pseudomonas stutzeri* strain 9)	Triton X-100 (biological system, *Achromobacter sp.* 4(2010))
*Γ* ^*∞*^	mol/m^2^	2.77 · 10^−6^	2.95 · 10^−6^	1.23 · 10^−6^
*A*_min_	m^2^	6.0 · 10^−19^	5.63 · 10^−19^	1.35 · 10^−18^
−Δ*G*_ads_	kJ/mol	38.18	17.5	19.3

*Γ* ^*∞*^ surface excess at the saturated interface, *A*_*min*_ minimum surface occupied by statistical molecule, *−ΔG*_*ads*_ Gibbs free energy of adsorption

What is more, for this system, the lowest minimum surface occupied by a statistical molecule in the adsorption layer was determined (*A*_min_ = 5.63 10^−19^). This means that Triton X-100 in *P. stutzeri* strain 9 system was more densely arranged at saturated water/air interface. Moreover, a surface excess at the saturated interface was also lowered.

### Analysis of fatty acids composition

A hydrophobic nature and low water solubility of diesel oil results in a limited availability of this carbon source for microorganism. Substances which can overcome such access issues are surfactants. They may intensify the biodegradation of diesel oil through its entrapment in surfactant micelles and in consequences mobilization of hydrophobic compounds. Intensification of a diesel oil biodegradation may also be connected with surfactant-induced changes in surface properties of bacterial cell (Wyrwas et al. 2011). These changes include the differences in a composition of fatty acids that are essential structural components of bacterial cell membranes, regulating their stability and fluidity (Mrozik et al. 2004, 2007; Sotirova et al. 2009).

**Fig. 6:**
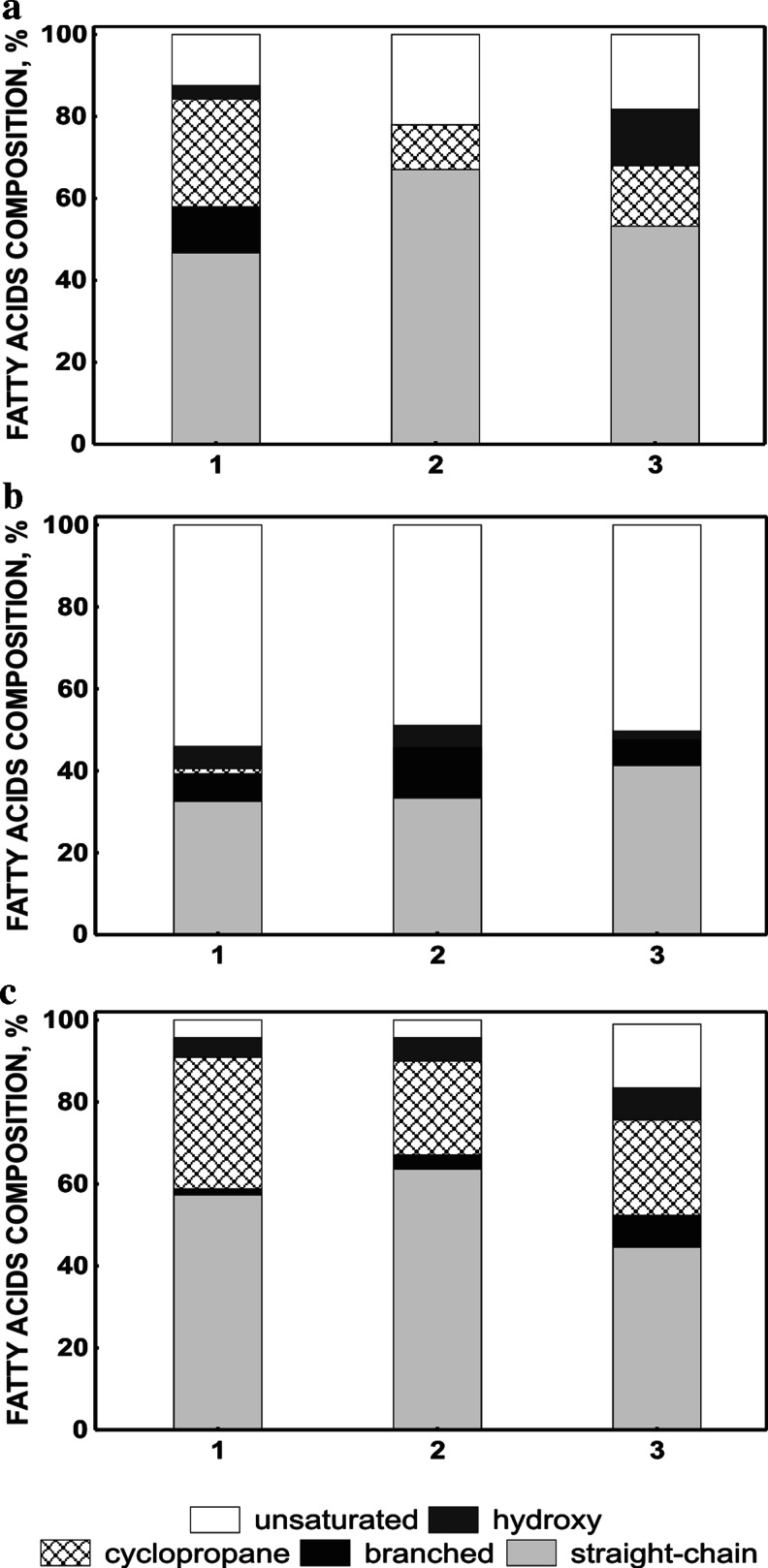
Proportions of fatty acids in *Achromobacter* sp. 4(2010) (**a**), *Pseudomonas stutzeri* strain 9 (**b**) and *Rahnella* sp. strain EK12 (**c**) growing on mineral salt medium supplemented with diesel oil (*1*), Triton X-100 (*2*), and both (*3*). Class of hydroxyl fatty acids contains additionally the branched hydroxyl fatty acids

To determine the effect of diesel oil and/or Triton X-100 on whole cell-derived fatty acids profiles of *Achromobacter* sp. 4(2010), *P. stutzeri* strain 9, and *Rahnella* sp*.* strain EK12 cultured on mineral salt medium supplemented with diesel oil, Triton X-100 or both were compared. Figure 6 presents the total percentage of the fatty acids and shows the compositional changes during growth of tested strains in the presence of diesel oil and/or Triton X-100. For the interpretation of diesel oil and/or Triton X-100 impact on bacteria, the identified fatty acids were grouped into two major classes. The first class included saturated fatty acids, which were additionally divided into four sub-classes: straight-chain, hydroxy, cyclopropane and branched fatty acids. The second class comprises unsaturated fatty acids.

For *Achromobacter* sp*.* 4(2010) and *Rahnella* sp*.* strain EK12 grown on mineral salt medium with diesel oil as a carbon and energy source, the content of saturated fatty acids was the highest (87.5 ± 1.6 % and 95.7 ± 0.1 % of total saturated fatty acids, respectively). In contrast, *P. stutzeri* strain 9 grown in the same arrangement revealed a lower amount of the saturated fatty acids (46.0 ± 3.2 %). The reaction of *Achromobacter* sp*.* 4(2010) and *Rahnella* sp*.* strain EK12 to the growth on Triton X-100 was an increase of straight-chain fatty acids amount (67.1 ± 0.4 % and 63.6 ± 1.3 %, respectively). Triton X-100 treatment of *P. stutzeri* strain 9 caused an increase of the branched fatty acids (from 6.7 ± 2.1 to 12.3 ± 0.9 %). Similar trends were observed by Mrozik et al. (2004) for *P. stutzeri*, *P. vesicularis* and *P. putida* grown on naphthalene. Such analogy might be explained by a similar effect of naphthalene and Triton X-100 (both possessing the aromatic rings in their molecules) on the bacterial cell. Presumably, the higher amount of branched fatty acids that was found in these systems appeared to be necessary for keeping the proper membrane stability (Mrozik et al. 2005).

Reaction of *Achromobacter* sp. 4(2010) grown on diesel oil and Triton X-100 was mainly linked to the higher level of hydroxyl fatty acids (13.8 ± 0.9 %). In contrast, *P. stutzeri* strain 9 and *Rahnella* sp*.* strain EK12 grown on the same substrates displayed an increase in straight-chain (41.3 ± 1.2 %) and unsaturated (16.6 ± 0.6 %) fatty acids, respectively. As a consequence, the changes in the saturated/unsaturated fatty acid ratio for *Rahnella* sp. strain EK12 were observed. In the presence of diesel oil and Triton X-100, this ratio was lower in comparison to monosubstrate systems (Online Resource 1).

Cyclopropane fatty acids have been known as compounds that stabilize membrane lipids and assist in tolerance towards disturbance (Denich et al. 2003; Mrozik et al. 2007). Surprisingly, the presence of Triton X-100 and Triton X-100-diesel oil mixture in the medium with *Achromobacter* sp. 4(2010), *P. stutzeri* strain 9 or *Rahnella* sp*.* strain EK12 resulted in a decrease in cyclopropane fatty acids 17:0 *cyclo* and 19:0 *cyclo ω*8*c* (Online Resource 1).

Broadly speaking, the degree of saturation/unsaturation and profile of fatty acids of bacterial membrane lipids result from reaction of bacteria on the presence of toxic compounds (Donato et al. 1997). However, results obtained by our research group and some other authors indicate that profile of fatty acids depends on the features of bacterial strains as well (Fang et al. 2004; Mrozik et al. 2007).

### Determination of hexadecane monooxygenase and catechol 2,3-dioxygenase

The performed assays of hexadecane monooxygenase and catechol 2,3-dioxygenase activity have proven that the three tested strains are able to produce both enzymes; nonetheless, they differ significantly in their activities (Table 3). The production of these two types of enzymes is justified by the complexity of diesel oil being a mixture of a wide range of hydrocarbons among which alkanes and aromatics play the most important role. The degradation pathway of alkanes starts with the primary attack of monooxygenases which results in the production of an alcohol that is next transformed within other enzymatic reactions. Likewise, the degradation of aromatics is started by the reaction of dioxygenases where two atoms of oxygen are incorporated to the ring leading to the formation of catechol (or its derivatives, depending on the substrate taken into the reaction) with a subsequent cleavage of the aromatic ring as a result (Sanakis et al. 2003).

**Table 3:** Activity of catechol 2,3-dioxygenase and hexadecane monooxygenase in the presence of Triton X-100

Enzyme	Specific activity (U mg^−1^ of protein) of bacterial strains		
	*Achromobacter* strain 4(2010)		
	A + T	A + DO	A + DO + T
Catechol 2,3-dioxygenase	0.0505 ± 0.0030	0.8689 ± 0.0353	1.0081 ± 0.0050
Hexadecane monooxygenase	0.0010 ± 0.0002	0.0034 ± 0.0001	0.0038 ± 0.0002
	*Rahnella* sp. strain EK12		
	R + T	R + DO	R + DO + T
Catechol 2,3-dioxygenase	0.2643 ± 0.0034	0.5461 ± 0.0105	1.1313 ± 0.0038
Hexadecane monooxygenase	0.0075 ± 0.0009	0.0089 ± 0.0007	0.0099 ± 0.0011
	*Pseudomonas stutzeri* strain 9		
	P + T	P + DO	P + DO + T
Catechol 2,3-dioxygenase	0.0880 ± 0.0052	1.2151 ± 0.2398	0.7195 ± 0.0003
Hexadecane monooxygenase	0.0174 ± 0.0017	0.0375 ± 0.0019	0.0174 ± 0.0017

A + T—the system containing *Achromobacter* strain 4(2010) and Triton X-100

A + DO—the system containing *Achromobacter* strain 4(2010) and diesel oil

A + DO + T—the system containing *Achromobacter* strain 4(2010), diesel oil and Triton X-100

Other samples were named analogously: *R Rahnella* sp. strain EK12, *P Pseudomonas stutzeri* strain 9

Although the presence of the two mentioned enzymes was detected, the dioxygenases revealed nearly ten times higher activity in all systems. This might indicate that either all strains have a higher ability to degrade aromatic hydrocarbons in general or that the dioxygenase expression is strongly related to the presence of diesel oil. Presumably, although the degradation pathway of aromatics is also possible through the stage catalysed by monooxygenases (for example toluene monooxygenase according to Mars et al. (1999)), the results indicate that the petroleum aromatic hydrocarbons are likely degraded through the meta-cleavage pathway (Mars et al. 1997) by all tested strains.

Furthermore, the comparison of the activities of enzymes in the presence of Triton X-100 in all tested systems and those noticed in the presence of diesel oil or its mixtures displayed that the surfactant did not inhibit the activities of the enzymes; however, it was also not a substance that could induce the production of the enzymes itself. The significant changes between the activities of both monooxygenases and dioxygenases with diesel oil and Triton X-100, diesel oil system, clearly showed the increase in the activities of two enzymes (Table 3). What is more, the highest activities of the enzymes were observed when considering the mixture of diesel oil and Triton X-100 in the bacterial cell cultivation. Interestingly, the only exception from this rule was the activity of catechol 2,3-dioxygenase in the system named P + DO + T, (meaning *P. stutzeri* strain 9 in diesel oil and surfactant arrangement); in this case, the enzyme activity was lower than in the absence of Triton X-100. This curious observation seems to draw the attention to the structure of enzyme. It could be possible that there are two competitive reactions taking place at the same time—between the enzyme and the surfactant or the enzyme and some aromatics from diesel oil. Such conclusion happens to find its proof when examining the results of Triton X-100 degradation. These two (or maybe more) reactions may lead to lowering of the actual activity of the enzyme. On the other hand, based on the literature reports considering the influence of surfactants on cell surface properties (Kaczorek et al. 2013b; Sałek et al. 2013), it is possible that some of the changes in the surface properties could have taken place. According to Li and Zhu (2012), who tested the biodegradation of polycyclic aromatic hydrocarbons (PAH), the bacterial uptake of these is possible due to interfacial processes in the cell surface. The alteration of the cell membranes could have an impact of the release of the enzyme and/or its activity.

## Conclusions

The obtained results indicate that Triton X-100 could be used as a surface active agent supporting the process of diesel oil biodegradation. Furthermore, the surfactant turned out to be biodegradable by all tested strains. The highest diesel oil biodegradation by *P. stutzeri* strain 9 was related to the hydrophilic surface properties of its cells. As for the enzymes, Triton X-100, diesel oil or their mixtures in the tested microbial systems demonstrated that the surfactant did not negatively affect the activities of the enzymes; nonetheless, it did not induce the production of the enzymes either.

## Electronic supplementary material

Below is the link to the electronic supplementary material.

Online Resource 1 (DOC 106 kb)
