# Supplementary material for: Bacterial properties changing under Triton X-100 presence in the diesel oil biodegradation systems: from surface and cellular changes to mono- and dioxygenases activities
Source: Environ Sci Pollut Res Int. 2014 Oct 8;22(6):4305–15. doi: 10.1007/s11356-014-3668-z (PMC4342841; doi:10.1007/s11356-014-3668-z)
Supplement: Supplementary file 1 — (DOC 106 kb) [file 11356_2014_3668_MOESM1_ESM.doc]

**Online Resource 1** Percentage of total fatty acids of *Achromobacter* strain 4 growing on mineral salt medium supplemented with diesel oil (1), mineral salt medium supplemented with Triton X-100 (2), mineral salt medium supplemented with Triton X-100 and diesel oil (3), *Pseudomonas stutzeri* strain 9 growing on mineral salt medium supplemented with diesel oil (4), mineral salt medium supplemented with Triton X-100 (5) and mineral salt medium supplemented with Triton X-100 and diesel oil (6), *Rahnella* sp. strain EK12 growing on mineral salt medium supplemented with diesel oil (7), mineral salt medium supplemented with Triton X-100 (8) and mineral salt medium supplemented with Triton X-100 and diesel oil (9)

| Fatty acids | % of total fatty acids | | | | | | | | |
| --- | --- | --- | --- | --- | --- | --- | --- | --- | --- |
|  | 1 | 2 | 3 | 4 | 5 | 6 | 7 | 8 | 9 |
| *Saturated* |  |  |  |  |  |  |  |  |  |
| 10:0 | - | - | - | 2.09±2.15 | - | - | - | - | - |
| 10:0 2OH | - | - | - | - | - | 2.18±0.5 | - | - | - |
| 10:0 3OH | 0.27±0.01 | - | - | - | 2.28±0.27 | - | - | - | 1.12±0.03 |
| 11:0 | - | - | - | - | - | - | - | 0.75±0.07 | 0.28±0.02 |
| 11:0 3OH | - | - | - | - | - | - | - | - | - |
| 11:0 *iso* | 0.14±0.02 | - | - | - | 2.25±0.45 | - | - | - | - |
| 11:0 *anteiso* | - | - | - | - | - | - | - | - | - |
| 11:0 *iso* 3OH | - | - | - | - | - | - | - | - | 0.45±0.02 |
| 12:0 | 6.10±0.25 | 6.87±0.24 | - | 6.20±0.90 | 6.20±1.82 | - | 11.08±0.16 | 12.59±1.64 | 8.21±0.15 |
| 12:0 2OH | 1.39±0.27 | - | 13.8±0.96 | - | - | - | 2.07±0.19 | 2.45±0.07 | 1.65±0.40 |
| 12:0 3OH | 0.33±0.04 | - | - | 3.35±0.58 | 3.14±0.45 | - | 0.38±0.04 | 0.58±0.03 | 0.96±0.49 |
| 12:0 *iso* | - | - | - | - | - | - | - | 0.48±0.04 | - |
| 12:0 *iso* 3OH | - | - | - | - | - | - | - | - | - |
| 13:0 | - | - | - | - | - | - | - | - | - |
| 13:0 2OH | - | - | - | - | - | - | - | 0.36±0.04 | 0.70±0.03 |
| 13:0 *iso* | - | - | - | - | - | - | - | - | - |
| 13:0 *anteiso* | 0.51±0.07 | - | - | - | - | - | - | - | - |
| 13:0 *iso* 3OH | - | - | - | - | - | - | - | - | - |
| 14:0 | 4.11±0.21 | - | - | 2.03±0.25 | 2.86±0.09 | 7.36±0.30 | 6.02±0.58 | 6.20±0.18 | 4.92±0.25 |
| 14:0 2OH | 0.68±0.14 | - | - | - | - | - | 1.31±0.04 | 1.46±0.08 | 1.34±0.33 |
| 14:0 *iso* | - | - | - | - | - | - | - | - | - |
| 14:0 *anteiso* | 0.47±0.03 | - | - | - | - | - | 0.12±0.02 | - | - |
| 15:0 3OH | - | - | - | - | - | - | - | - | 0.33±0.11 |
| 15:0 *iso* | 1.07±0.07 | - | - |  |  | - | - | - | - |
| 15:0 *anteiso* | 2.89±0.25 | - | - | 2.28±0.03 | 3.13±0.49 | 2.21±0.24 | - | - | 2.56±0.10 |
| 16:0 | 33.77±2.04 | 54.17±1.01 | 53.22±2.36 | 22.27±3.08 | 22.67±0.25 | 25.01±0.72 | 36.63±1.13 | 40.03±1.25 | 28.48±0.73 |
| 16:0 2OH | 0.20±0.04 | - | - | - | - | - | 0.24±0.01 | - | 0.25±0.01 |
| 16:0 3OH | 0.39±0.09 | - | - | - | - | - | 0.74±0.03 | 0.81±0.07 | 1.04±0.06 |
| 16:0 *iso* | 0.95±0.10 | - | - | 0.55±0.01 | - | - | - | - | - |
| 16:0 *anteiso* | - | - | - | 1.30±0.31 | - | 4.04±0.68 | - | - | 2.23±0.06 |
| 17:0 | 0.39±0.10 | - | - | - | - | - | 1.07±0.05 | 1.39±0.03 | 0.72±0.08 |
| 17:0 *iso* | 0.44±0.03 | - | - | - | - | - | - | - | - |
| 17:0 *anteiso* | 3.74±0.21 | - | - | - | - | - | - | - | 2.12±0.03 |
| 17:0 *cyclo* | 20.86±3.60 | 10.92±2.26 | 14.76±1.38 | - | - | - | 27.3±1.16 | 16.40±0.55 | 19.36±1.87 |
| 18:0 | 2.36±3.60 | 6.01±0.81 | - | 2.07±0.02 | 1.63±0.23 | 8.92±0.39 | 2.53±0.15 | 2.65±0.31 | 1.89±0.37 |
| 19:0 *iso* | 0.99±0.13 | - | - | - | - | - | 1.34±0.05 | 2.98±1.65 | 0.87±0.01 |
| 19:0*cyclo w*8*c* | 5.45±0.81 | **-** | - | 1.27±0.19 | - | - | 4.83±0.35 | 6.55±2.21 | 3.88±0.36 |
| *Unsaturated* |  |  |  |  |  |  |  |  |  |
| 12:1 3OH | - | - | - | - | - | - | - | - | - |
| 13:1*w*12*c* | - | - | - | - | - | - | - | - | 0.25±0.05 |
| 15:1 *isoF* | - | - | - | - | - | - | - | - | 0.26±0.02 |
| 15:1 *isoG* | - | - | - |  | - | - | - | - | 0.51±0.04 |
| 15:1*w*6*c* | - | - | - | - | - | - | - | - | - |
| 16:1*w5c* | - | - | - | - | - | - | - | - | 0.45±0.03 |
| 16:1*w*9*c* | 0.47±0.05 | - | - | - | - | 4.96±0.35 | 0.42±0.02 | - | 0.36±0.10 |
| 16:1*w*7*c*/16:1*w*6*c* | 1.56±0.46 | - | - | 18.47±2.99 | 23.51±0.61 | - | 1.20±0.06 | - | 1.53±0.08 |
| 17:1*w*7*c* | - | - | - | - | - | - | 0.39±0.06 | 0.77±0.03 | - |
| 17:1*isoI/anteisoB* | - | - | - | - | - | - | - | - | - |
| 17:1 *iso* *w*9*c* | - | - | - | - | - | - | - | 0.88±0.02 | 0.31±0.05 |
| 18:1 *w*6*c* | - | - | - | - | - | - | - | 1.54±0.09 | - |
| 18:1 *w*7*c* | 2.43±0.09 | 5.57±0.68 | - | 26.31±0.31 | 21.57±1.21 | - | 2.32±0.15 | 1.13±0.06 | 2.96±0.15 |
| 18:1 *w*9*c* | 3.56±0.50 | 16.46±1.19 | 18.21±1.93 | 6.19±0.34 | 3.86±0.23 | 40.18±1.28 | - | - | 6.69±0.19 |
| 19:1*w*6*c* | 1.71±0.16 | - | - | - | - | - | - | - | - |
| 19:1*w*11*c* | 0.23±0.01 | - | - | - | - | - | - | - | 0.09±0.04 |
| 20:1 *w*7*c* | - | - | - | - | - | - | - | - | - |
| 18:2 *w*6,9*c* | 2.52±0.44 | - | - | 3.05±0.70 | - | 5.14±0.36 | - | - | 3.15±0.60 |
| *Other* | 0.00±0.00 | 0.00±0.00 | 0.01±0.00 | 0.00±0.00 | 0.00±0.00 | 0.00±0.00 | 0.00±0.00 | 0.01±0.00 | 0.01±0.00 |
| *Sat./Unsat. ratio* | 7.01±1.11 | 3.54±0.4 | 4.49±0.6 | 0.85±0.11 | 1.04±0.07 | 0.99±0.05 | 22.11±0.45 | 22.17±0.93 | 5.04±0.22 |
